# Supplementary material for: Predictors of discharge disposition and mortality following hospitalization with SARS-CoV-2 infection
Source: PLoS One. 2023 Apr 13;18(4):e0283326. doi: 10.1371/journal.pone.0283326 (PMC10101512; doi:10.1371/journal.pone.0283326)
Supplement: S1 Table — (DOCX) [file pone.0283326.s001.docx]

Supplemental Table 1: New Onset SARS-COV-2 symptoms on presentation by discharge disposition using Fisher’s exact test.

|  | Total | | Home | IRF | SNF | LTACH | Hospice/Expired | p-value |
| --- | --- | --- | --- | --- | --- | --- | --- | --- |
|  | N=5,593 | N=4,098 | | N=115 | N=747 | N=43 | N=590 |  |
| New Body aches | 2.2% | 2.2% | | 2.4% | 1.7% | 14.3% | 2.0% | 0.019 |
| New Sore throat | 0.9% | 1.1% | | 2.4% | 0.7% | 0.0% | 0.0% | 0.061 |
| New Rhinitis/congestion | 2.1% | 2.1% | | 0.0% | 2.7% | 0.0% | 1.8% | 0.595 |
| New Nausea/Vomiting | 4.6% | 4.6% | | 2.4% | 5.4% | 7.1% | 3.9% | 0.556 |
| New Diarrhea | 2.4% | 2.2% | | 1.2% | 2.6% | 0.0% | 3.4% | 0.570 |
| New Fatigue | 4.8% | 4.6% | | 4.9% | 5.4% | 10.7% | 4.5% | 0.480 |
| New Dyspnea | 5.8% | 5.6% | | 4.9% | 6.3% | 14.3% | 5.9% | 0.363 |
| New Cough | 6.2% | 6.2% | | 6.1% | 6.3% | 7.1% | 6.3% | 0.987 |
| New Joint pain | 1.0% | 1.0% | | 1.2% | 1.0% | 0.0% | 1.4% | 0.754 |
| New Chest Pain | 2.7% | 2.8% | | 0.0% | 2.4% | 3.6% | 2.9% | 0.535 |
| New Brain Fog | 0.0% | 0.0% | | 0.0% | 0.0% | 0.0% | 0.0% | 1.000 |
| New Depression | 2.1% | 1.9% | | 2.4% | 2.4% | 3.6% | 2.9% | 0.369 |
| New Muscle Pain | 1.5% | 1.6% | | 2.4% | 0.3% | 14.3% | 1.4% | 0.0002 |
| New Headache | 3.4% | 3.5% | | 6.1% | 2.7% | 7.1% | 2.9% | 0.280 |
| New Fever | 5.6% | 5.5% | | 8.5% | 5.8% | 14.3% | 4.8% | 0.189 |
| New Palpation | 4.7% | 4.4% | | 4.9% | 5.1% | 17.9% | 5.4% | 0.042 |
| New Rash | 2.4% | 2.4% | | 1.2% | 2.6% | 7.1% | 2.7% | 0.453 |
| New Hair loss | 0.0% | 0.0% | | 0.0% | 0.0% | 0.0% | 0.0% | 1.000 |
| New Loss of Smell/taste | 0.6% | 0.8% | | 1.2% | 0.2% | 0.0% | 0.0% | 0.103 |
| New Insomnia | 0.9% | 0.7% | | 2.4% | 1.0% | 3.6% | 1.1% | 0.112 |
| New Difficulty thinking | 0.0% | 0.0% | | 0.0% | 0.0% | 0.0% | 0.0% |  |
| New Difficulty with Memory | 0.4% | 0.2% | | 1.2% | 1.0% | 0.0% | 0.5% | 0.033 |
| New Anxiety | 1.7% | 1.7% | | 0.0% | 2.0% | 3.6% | 0.9% | 0.327 |
