# Supplementary material for: Predictors of discharge disposition and mortality following hospitalization with SARS-CoV-2 infection
Source: PLoS One. 2023 Apr 13;18(4):e0283326. doi: 10.1371/journal.pone.0283326 (PMC10101512; doi:10.1371/journal.pone.0283326)
Supplement: Attachment — Submitted filename: Response to Review PLOS ONE Final (3).docx [file pone.0283326.s002.docx]

Response to Review

We thank the reviewers and the editorial staff for their thoughtful critique of our work. Reviewers found the topic important and the findings hypothesis generating to support future studies. We have revised the manuscript to address the concerns/suggestions raised.  Below are reviewer’s specific suggestions followed by our responses.

**Comments to the Author**

**1. Is the manuscript technically sound, and do the data support the conclusions?**

**The manuscript must describe a technically sound piece of scientific research with data that supports the conclusions. Experiments must have been conducted rigorously, with appropriate controls, replication, and sample sizes. The conclusions must be drawn appropriately based on the data presented.**

**Reviewer #1: Partly**

**Reviewer #2: Partly**

**2. Has the statistical analysis been performed appropriately and rigorously?**

**Reviewer #1: Yes**

**Reviewer #2: Yes**

**3. Have the authors made all data underlying the findings in their manuscript fully available?**

**The** [**PLOS Data policy**](http://www.plosone.org/static/policies.action#sharing) **requires authors to make all data underlying the findings described in their manuscript fully available without restriction, with rare exception (please refer to the Data Availability Statement in the manuscript PDF file). The data should be provided as part of the manuscript or its supporting information or deposited to a public repository. For example, in addition to summary statistics, the data points behind means, medians and variance measures should be available. If there are restrictions on publicly sharing data—e.g., participant privacy or use of data from a third party—those must be specified.**

**Reviewer #1: No**

**Reviewer #2: Yes**

**4. Is the manuscript presented in an intelligible fashion and written in standard English?**

**PLOS ONE does not copyedit accepted manuscripts, so the language in submitted articles must be clear, correct, and unambiguous. Any typographical or grammatical errors should be corrected at revision, so please note any specific errors here.**

**Reviewer #1: Yes**

**Reviewer #2: Yes**

**5. Review Comments to the Author**

**Please use the space provided to explain your answers to the questions above. You may also include additional comments for the author, including concerns about dual publication, research ethics, or publication ethics. (Please upload your review as an attachment if it exceeds 20,000 characters)**

Editor

We now have two reviews of your study. You will see that both reviewers raised a number of strengths but also very clearly described their concerns with this study. Please note that one of the reviewers raised important issues related to the circulating variant(s) and vaccine uptake at the time of your study and the need to better contextualize your results within this. As well, both reviewers raised important points regarding the analysis and the need for a clearer presentation - and rationale - for certain aspects.

**Reviewer #1: In this multicenter retrospective study, the authors describe and compare discharge dispositions following hospitalization among patients infected with SARS-CoV-2 and influenza. The authors find significant differences in the number of patients discharged to post-acute care (PAC) with a higher proportion of SARS-CoV-2 patients discharged to PAC, as well as longer length of hospitalization and higher mortality among patients with SARS-CoV-2. The authors also investigate associations between various clinical and laboratory parameters and discharge to PAC for patients with SARS-CoV-2. The strengths of the study include the large sample size as well as linking of patient data to state death certificates to characterize out of hospital death. My comments regarding this study are summarized below:**

**Major Comments:**

**1. Can the authors justify why these laboratory parameters were chosen to be included? Some of the variables such as CRP and albumin are more intuitive as markers of inflammation and nutritional status. Others are less so. For instance, why are blood type and Rh positive include in the analysis?**

Earlier studies have had specific questions about the association between blood types and susceptibility to developing severe infection based on blood types. found that ABO polymorphism was associated with increased susceptibility to infection with SARS-CoV-1 through inhibition of anti-A antibodies of uptake to the ace converting enzyme receptors-2 (Cheng Y, Cheng G, Chui CH. ABO blood group and susceptibility to the severe acute respiratory syndrome. *JAMA.* 2005; 293:1450–1451). Some studies had concluded that antigen A on blood type A antigen found in the lungs of people with blood type A may provide insight into the potential link between blood group A and COVID-19 infection (Wu, S.-C., Arthur, C.M., Wang, J., Verkerke, H., Josephson, C.D., Kalman, D., Roback, J.D., Cummings, R.D., Stowell, S.R., 2021. The SARS-CoV-2 receptor-binding domain preferentially recognizes blood group A. Blood Advances 5, 1305–1309. doi:10.1182/bloodadvances.2020003259). Given the questions being asked, we included blood types as a variable to analyze if they made an impact on the severity of the infection and hospitalization/post-acute care needs. We have included this justification in the discussion part of the manuscript in our revision.

**2. It is challenging to assess the clinical relevance of the differences found in laboratory parameters for hemoglobin, white blood cell count, platelets, etc. (Table 3). Most of the reported medians and interquartile ranges fall within the normal range of values for these variables. The authors could consider presenting these as categorical variables with cut-offs for low, normal, and high values. As it stands, it seems difficult to conclude that differences in WBC, where the medians range from 6 – 8, suggest significantly different states of inflammation.**

We agree that there were subtle differences in the values of the certain laboratory values in this patient population. The point made by the reviewers is an important point and will add clarity to the readers. In this revision, we categorized labs based on normal values as high, normal, and low, however, for the regression models, we used values as continuous and imputed values.

**3. For the multinomial logistic regression, how were variables of interest selected for inclusion in the model? Were these selected a-priori?**

Thank you for this important question. The variables in the logistic regression were based on previously published variables of interest. The values were selected a-priori and selection were based on the interest of these factors/variables to COVID severity and subsequent hospitalization. One study published in PLOS ONE in 2021 by Yang showed that pre-admission leucocytes were associated with an increased mortality rate in COVID patients (1.	Yang, L., et al., *Risk factors for predicting mortality of COVID-19 patients: A systematic review and meta-analysis.* PLoS One, 2020. **15**(11): p. e0243124). In this study, pretreatment LDH, Procalcitonin, D-Dimer, and ferritin levels were also associated with increased mortality. Platelets were added in our study due to the increased coagulopathy complications seen in COVID 19 papers.

**4. The rationale for evaluating presenting symptoms and association with discharge disposition is not entirely clear. Do you hypothesize that presenting symptoms may reflect the severity of disease at presentation or potential extrapulmonary involvement? For instance, fever or body aches may suggest a more systemic inflammatory response.**

We thank the reviewer for both asking the question and answering the question so eloquently. We will add this clarification in our discussion. Association between symptoms and severity of the COVID infection has been a matter of interest. Yang et al reported that symptoms of dyspnea, chest tightness, hemoptysis, expectoration, and fatigue were associated with increased mortality in COVID19 patients[(Yang et al. 2020)](https://paperpile.com/c/Zm2vBJ/5QBG).  (Yang, L., et al., Risk factors for predicting mortality of COVID-19 patients: A systematic review and meta-analysis. PLoS One, 2020. **15**(11): p. e0243124)

**5. The authors note that fewer Black and Asian patients are discharged to PACs compared to home. Is there literature regarding racial/ethnic disparities in hospital discharge disposition accounting for the severity of illness and co-morbidities? This finding is worth noting in the discussion.**

This is an excellent comment that we should have attended to, while there have been many studies looking at the rate of hospitalization of various populations following a positive COVID, there has been a paucity of studies looking at the rehabilitation needs and disparities of access in the Black and Asian patients to discharge to rehabilitation inpatient units following hospitalization. Our study is unique to find that fewer Blacks and Asians were discharged to PACs and has been included in the discussion.

**6. Is data available regarding where patients are coming from on hospital admission? (E.g., residing at home vs. SNF). Certainly, early in the pandemic, many SNFs had large COVID outbreaks. This may impact the interpretation of results in the sense that some patients may be returning to SNF on discharge.**

Agree that this is an important part of the data which should have been included. We corrected this deficiency. We went back to our data again and found that 95% of the patients presented from their homes, 3.6% came from the SNF, and 1.4% were transferred from another hospital.  This has been added to the Results section line 218.

**7. What is the minimum duration of observation between hospital discharge and out-patient death? In other words, were death certificates updated 3 months after the last discharge, 6 months etc.?**

The state death certificates are updated daily, the system reruns the database daily, and any patient who is deceased is captured.

**Minor Comments:**

**1. In the introduction, the significance is framed around the current influenza season. Given that the influenza season typically peaks in February or March in the US, the authors should consider updating some of the phrasing here.**

Thank you for the comment, we agree to update this information.

**2. Why does available data stop in November 2020 for influenza patients? As opposed to June 2021 for SARS-CoV-2 patients.**

The influenza data were from patients admitted with the primary diagnosis of ‘influenza’, the acute care data specifically for COVID was started in December 2019, and the influenza data did not go beyond November 2020.  See page 70 under section “participants”.

**3. Page 11-12: It appears that there is duplicated text in the methods for the paragraphs starting with “State death certificates were linked”.**

We reviewed the stated pages, and confirmed the duplication, we applaud the reviewers for their excellent review of the manuscripts. This was corrected.

**4. For clarity throughout the paper, I would suggest specifying that expired refers to in-hospital death throughout the text. Similarly, would clarify what “Mortality” in Tables 2 and 3 refers to. Is this combined in and out of hospital death?**

We have clarified that ‘Died in hospital (DH)’ refers to in-hospital death in line 120 as per reviewer comment, Mortality refers to out of hospital deaths based on state death certificates, see line 121. This is referred to as ‘Mortality” in Tables 2 and 3.

**5. Table 3 - is there an error in median ICU LOS? Should this be presented as the median (IQR) of ICU LOS for patients who were admitted to the ICU in each respective category?**

The ICU LOS is represented as median and IQR.

**6. Table 3 – There is no p-value for CRP.**

Thank you for bringing this to our attention, we corrected this running the analysis and the p-value was noted to be significant

**7. Please include in the supplement which explanatory variables were excluded due to missingness and which were imputed. Missingness of 44% is quite high.**

Please see page 120. We built a model of known co-variates that have been associated with specific outcomes in COVID19, and these were included apiori. We then calculated the missingness of the co-variables and imputed the missingness of the variables.

**8. This may be a technical issue, but several of the mentioned supplemental** tables and flow charts are not available on my review.

We apologize and will correct that and make sure the supplements are available for your review.

**9. There are a few typos in the manuscript – E.g., Page 17 “between the age of 65 and 8418”.**

Thank you for the correction, it was corrected to 84 years based on the study (Jeganathan, N., Grewal, S. & Sathananthan, M. Comparison of Deaths from COVID-19 and Seasonal Influenza in the USA. *Lung* **199**, 559-561, doi:10.1007/s00408-021-00468-0 (2021)). See page 309.

**Reviewer #2: Thank you for the opportunity to read your work.**

**The question asked seems relevant to me: what are the PAC needs for patients with Covid? Can we identify the predictive factors for final discharge to CAP?**

**Integrating patients with Influenza during other periods/years complicates the work and makes the results less readable. This question seems to me to belong to another study.**

**Introduction**

**The issue of SARS-Cov-2 variants is major. We know that the severity of the disease is strongly linked to the identified variant and to the vaccination status of the patient. this should be discussed in the introduction. In your work the variants have not been identified and it seems to me that this may be a major weakness in the work. You should specify the distribution of variants during the study period, and the vaccination rates of the general population and the population at risk of developing severe forms.**

**Methods**

**Discharge to PAC is very variable and depends on local practices, the availability of places in these structures, modes of financing, a medical and probably also social culture in the place of the elderly, and the possibilities of maintaining homes for these patients.**

**I think it would have been interesting to describe the usual population (even admitted during the same period of study) admitted to your centers, those transferred to PAC, and describe the predictive factors of transfer.**

**The choice of influenza, as I can understand, is skewed if you haven't performed routine influenza screening in recent patients admitted for respiratory manifestations.**

**And the practices and the availability of places and PAC have certainly changed during the period of the study.**

**A flowchart of the patients included in the study should be presented. The tested, the admitted with SARS-Cov-2 and Influenza virus.**

**The severity of patients, their previous condition, and their condition at discharge are also variables to be assessed. Differences between centers and between age categories, etc., may also influence the final decision. This is not discussed in your introduction nor in the method.**

**What means hospice/expired? It is a composite endpoint?**

**Results**

**I have a little trouble understanding the care pathways: how many came from outpatient facilities and how many were diagnosed and probably immediately serious in the emergency room?**

**I think we need to reduce the text and better present the results in tables. The flu vs Covid comparisons make it difficult to understand the results.**

**Patients with SARS CoV 2 are more serious, more often polypathological, and older. Their hospital stay is more favorable, shorter and require less sheave. Can we compare them on the orientations at the exit?**

**In the prediction model, did you include patients with Influenza? If so, the Covid does not appear as a predictive factor?**

**Or is it only the identified factors that explain the observed differences?**

**Discussion**

**The first paragraph should state the strengths of your work and your main results, your most important messages.**

**The discussion on mortality (in the general population and in-hospital...), the clinical characteristics and the severity factors of Influenza make it difficult to understand the text.**

**I don't understand the third paragraph, due to the discussion of PAC needs of flu patients.**

**The fourth paragraph: I don't think you can really conclude that the bed needs of patients with SARS CoV 2 are higher than those of patients with Influenza. The context and overload of the healthcare system was not the same during the pandemic period as in previous years during periods of seasonal flu.**

**Logic would have wanted to discuss first the predictive value of CAP of the variables related to the terrain, then the clinical picture, then the biological parameters and clinical characteristics, then the variables related to severity then the variables related to the hospital stay.**

**A question: the transfer rates were the same in the 12 centers? Did you notice a center effect? This can be important in the discussion of center practices and habits.**

**You discuss inflammatory markers in Covid, but you haven't assessed C-reactive protein, a recognized severity marker in those patients. Could you be more precise?**

Agree that the CPR is an important and recognized marker and has been added in the revision with a significant p-value. In our analysis, high admission CRP was associated with discharge to PAC 73.0 (32.0-129.0) versus home (67.0 29.0-119.0) versus 117.0 (63.5-176.0) for those who died while hospitalized. This is noted to be significant.

**Conclusion**

**I am not sure that you can say that the PAC needs of patients admitted for SARS-Cov-2 are higher than those of patients admitted for Influenza.**

**I don't understand why you say the predictors of discharge to PAC are different...or the presentation in the text and tables is confusing.**

**Tables**

**Tables 1 and 2**

**Table 1 too short with too long presentation of results in Results. Table 1 and 2 should be merged and simplified.**

**Table 3**

**These results are very briefly discussed in the discussion. Is it really important to keep them?**

**Chart 4**

**This analysis only concerns patients with SARS-Cov-2 or does it include patients with Influenza?**

**I don't see Covid/influenza in the variables.**

Thank you for the comments. We agree, we have now removed the Table 1.

We have modified the results of the Table 3, merging the various post-acute care facilities into one outcome.

Table 4 is a regression analysis with discharge home as a base outcome. This has been discussed in detail. Influenza is not included in it, but the variants and breakthrough infections have been added.
