# Supplementary material for: Predictors of discharge disposition and mortality following hospitalization with SARS-CoV-2 infection
Source: PLoS One. 2023 Apr 13;18(4):e0283326. doi: 10.1371/journal.pone.0283326 (PMC10101512; doi:10.1371/journal.pone.0283326)
Supplement: Attachment — Submitted filename: Response to Reviewers PLOS ONE 2023.docx [file pone.0283326.s003.docx]

Reviewer's Responses to Questions

**Comments to the Author**

1. If the authors have adequately addressed your comments raised in a previous round of review and you feel that this manuscript is now acceptable for publication, you may indicate that here to bypass the “Comments to the Author” section, enter your conflict-of-interest statement in the “Confidential to Editor” section, and submit your "Accept" recommendation.

Reviewer #3: (No Response)

Reviewer #4: (No Response)

2. Is the manuscript technically sound, and do the data support the conclusions?

The manuscript must describe a technically sound piece of scientific research with data that supports the conclusions. Experiments must have been conducted rigorously, with appropriate controls, replication, and sample sizes. The conclusions must be drawn appropriately based on the data presented.

Reviewer #3: Yes

Reviewer #4: Yes

3. Has the statistical analysis been performed appropriately and rigorously?

Reviewer #3: Yes

Reviewer #4: I Don't Know

4. Have the authors made all data underlying the findings in their manuscript fully available?

The [PLOS Data policy](http://www.plosone.org/static/policies.action#sharing) requires authors to make all data underlying the findings described in their manuscript fully available without restriction, with rare exception (please refer to the Data Availability Statement in the manuscript PDF file). The data should be provided as part of the manuscript or its supporting information, or deposited to a public repository. For example, in addition to summary statistics, the data points behind means, medians and variance measures should be available. If there are restrictions on publicly sharing data—e.g. participant privacy or use of data from a third party—those must be specified.

Reviewer #3: Yes

Reviewer #4: Yes

5. Is the manuscript presented in an intelligible fashion and written in standard English?

PLOS ONE does not copyedit accepted manuscripts, so the language in submitted articles must be clear, correct, and unambiguous. Any typographical or grammatical errors should be corrected at revision, so please note any specific errors here.

Reviewer #3: Yes

Reviewer #4: Yes

6. Review Comments to the Author

Please use the space provided to explain your answers to the questions above. You may also include additional comments for the author, including concerns about dual publication, research ethics, or publication ethics. (Please upload your review as an attachment if it exceeds 20,000 characters)

Reviewer #3: This submission by Ikramuddin et al performed retrospective analysis on a cohort of 62,279 hospitalized SARS-CoV-2 positive patients. They investigated factors associated with either discharge home (-/+ Mortality), or to an inpatient post-acute setting (-/+ death) and those that died in hospital.

They found that discharge to PAC and in hospital death was associated with older age, higher EI, along with elevated CRP and D-dimer levels (factors associated with worse outcome in SARS-CoV-2 infection) compared to those that were discharged home.

I was invited as an additional reviewer and I was unable to see the tracked changes version of the manuscript, so I cannot comment on how the manuscript has changed since the previous version.

I may have missed something, but cannot see any mention of influenza patients and discharge to PAC or home, given that in the discussion the authors state that they wanted to investigate differences in PAC utilization.

The authors state that D-dimer is an inflammatory marker. D-dimer is a marker of coagulation activation and not inflammation, this needs to be clarified in the text.

Table 2 + 3 need to define cut-off values for "high, low and normal" for hemoglobin, WBC and plts.
Include units within the tables.

Response to the reviewer:

Agree with the reviewer that the influenza discharge disposition is lacking, we have added the table now as Table 7 which shows that 5.9% of the influenza patients were discharged to PAC and 0.6% died while being hospitalized.

Agree that the D dimer is not an inflammatory marker rather a marker coagulation activation. This was corrected in lines 231 and 808 to reflect it.

Table 2 and 3 were corrected to define values of normal, low and high of hemoglobin, platelets and white blood cells.

Reviewer #4: It is a very well written article. I do not have much comments in terms of improving the quality of the article.

7. PLOS authors have the option to publish the peer review history of their article ([what does this mean?](https://journals.plos.org/plosone/s/editorial-and-peer-review-process#loc-peer-review-history)). If published, this will include your full peer review and any attached files.


If you choose “no”, your identity will remain anonymous, but your review may still be made public.

No


**Do you want your identity to be public for this peer review?** For information about this choice, including consent withdrawal, please see our [Privacy Policy](https://www.plos.org/privacy-policy).

Reviewer #3: No

Reviewer #4: No
