# Supplementary material for: Predictors of discharge disposition and mortality following hospitalization with SARS-CoV-2 infection
Source: PLoS One. 2023 Apr 13;18(4):e0283326. doi: 10.1371/journal.pone.0283326 (PMC10101512; doi:10.1371/journal.pone.0283326)
Supplement: Attachment — Submitted filename: Response to Reviewers 2-8-2023.docx [file pone.0283326.s004.docx]

Re: Predictors of Discharge Disposition and Mortality following Hospitalization with SARS-CoV-2 Infection

Dear Editor and reviewers,

The reviewers had provided one comment to standardize the formatting of D-Dimer throughout the paper. I chose to use the standard as used by NIH as D-Dimer. This change was made in 18 different locations in the paper and has been thus corrected.

Thank you

Farha Ikramuddin

2/8/2023
